# A Clot and a Clue: When Pulmonary Embolism Leads to a Renal Diagnosis

**DOI:** 10.7759/cureus.94389

**Published:** 2025-10-12

**Authors:** Valter Duarte, Jéssica Krowicki, Miguel Martins, Telma Santos, Gorete Jesus

**Affiliations:** 1 Internal Medicine, Unidade Local de Saúde da Região de Aveiro, Aveiro, PRT; 2 Nephrology, Unidade Local de Saúde da Região de Aveiro, Aveiro, PRT

**Keywords:** hypoalbuminemia, nephrotic syndrome, primary membranous nephropathy, proteinuria, pulmonary embolism (pe)

## Abstract

Primary membranous nephropathy (PMN) is one of the leading causes of nephrotic syndrome (NS) in non-diabetic adults, potentially progressing to end-stage renal disease. The detection of specific antibodies, such as anti-phospholipase A2 receptor (anti-PLA2R), is useful for diagnosis, risk stratification, and monitoring therapeutic response. Clinical presentation is usually insidious, with edema, malaise, fatigue, and anorexia, but may be complicated by NS complications as thrombotic events. We present the case of a 45-year-old man with bilateral pleuritic chest pain and hemoptoic cough for the past week, and peripheral edema over six months. Contrast-enhanced chest computed tomography (CT) revealed bilateral pulmonary embolism (PE). Laboratory tests highlighted hypoalbuminemia (2.4 g/dL), nephrotic-range proteinuria (12 g/24h) with preserved renal function, and positive anti-PLA2R antibodies (80 RU/mL). Secondary membranous nephropathy was excluded and renal biopsy was deferred. Treatment with rituximab was initiated, resulting in analytical improvement and disappearance of anti-PLA2R antibodies at six months. Close monitoring of renal function and early initiation of conservative treatment are essential to prevent disease progression and complications of NS, with immunosuppressive therapy potentially required in selected cases.

## Introduction

Membranous nephropathy is an autoimmune disease and represents the second most common cause of nephrotic syndrome (NS) in adults after diabetic nephropathy [1-3], with a global incidence of 8-10 cases per 1 million individuals [2,3]. It is characterized by subepithelial deposition of immunoglobulins, leading to damage of the glomerular basement membrane and resulting in renal protein loss. In approximately 80% of cases, a specific etiological cause is not identified, while the remaining cases are associated with medications, other autoimmune diseases, infections, or malignancies [2-4].

It can occur at any age but is most prevalent between 35 and 40 years [2]. It usually presents with NS, predominantly characterized by proteinuria (often in the nephrotic range, >3.5 g/day), edema, hypoalbuminemia and hyperlipidemia. The onset tends to be insidious, typically with preserved renal function at the time of diagnosis. Around 30% of patients achieve complete remission of proteinuria, while another 30% may progress to end-stage renal disease within 10 years [1,2,4]. There is also an increased risk of thrombotic events (more commonly venous), especially linked to the severity of hypoalbuminemia and the duration of disease, particularly within the first six months [1,2,5].

The identification of disease-specific antibodies has increasingly reduced the need for renal biopsy, which, despite still being considered the diagnostic gold standard, is now reserved for patients with atypical progression, lack of treatment response or when secondary causes are suspected [1-3]. The antibody against the phospholipase A2 receptor (anti-PLA2R) is present in approximately 70% of patients with primary membranous nephropathy, and its measurement is used not only for diagnosis but also as a prognostic marker and indicator of treatment response [1-3]. Other antibodies are currently under investigation for similar purposes, some of which are associated with secondary forms of the disease [1-3].

Given the potentially benign course of the disease, most cases are initially managed with a period of surveillance and conservative treatment using angiotensin-converting enzyme inhibitors (ACEi) [1,2,4,6]. More recently, sodium-glucose cotransporter 2 inhibitors have been used to achieve a faster and more sustained reduction in proteinuria [1,7]. In patients at risk of progressive renal function deterioration and/or with severe complications of nephrotic syndrome, immunosuppressive therapy becomes necessary. The discovery of antibodies targeting podocytes has made B-cell depleting agents and calcineurin inhibitors a cornerstone of treatment, while glucocorticoids and cyclophosphamide are now generally reserved for more severe cases [1,2,8].

## Case presentation

We present a case of a 45-year-old man with no relevant past medical history who presented to the emergency department with bilateral pleuritic chest pain and productive hemoptoic cough with one week of evolution. He also reported bilateral lower limb edema for the past six months, partially responsive to furosemide. He had no family history of cancer or autoimmune diseases and no history of recent immobilization, infections or medication use.

**Figure 1:**
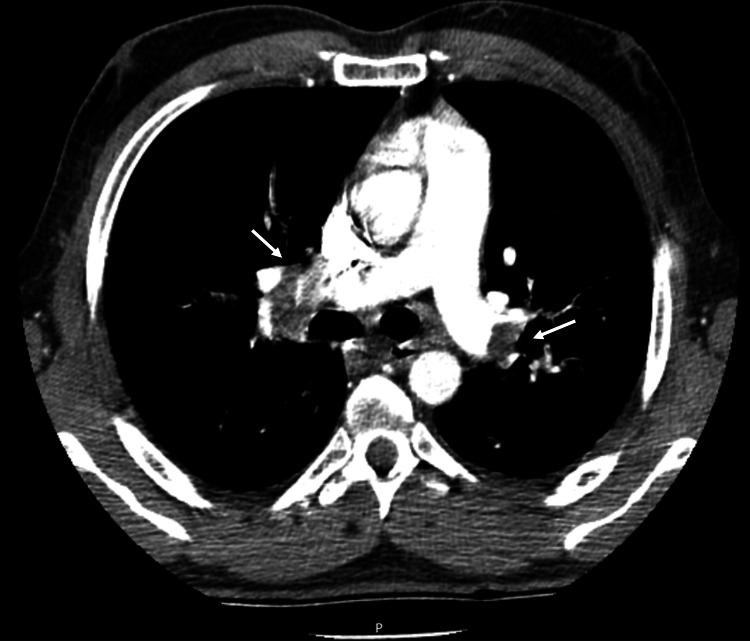
Thoracic contrast-enhanced computed tomography showing extensive pulmonary thromboembolism, with defects at bifurcation sites (white arrows), extending to lobar branches and to the inferior, middle, and lingular segmental branches.

On admission, he was afebrile, tachypneic with an oxygen saturation of 93% on room air, and had bibasilar crackles. He was normotensive (Blood pressure of 138/85 mmHg) and had sinus tachycardia (heart rate of 140 bpm) with bilateral lower limb edema. Initial blood tests showed leukocytosis (16,400 cells/µL), elevated C-reactive protein (6.26 mg/dL), markedly elevated D-dimers (38 µg/mL), and N-terminal pro-B-type natriuretic peptide (1146 pg/mL), with normal troponin levels. A thoracic contrast-enhanced computed tomography (CT) was performed confirming bilateral pulmonary embolism (PE) (Figure 1) without signs of right ventricular dysfunction on transthoracic echocardiogram. Anticoagulation with enoxaparin was initiated and the patient was admitted for monitoring and further workup.

Further investigation revealed an elevated erythrocyte sedimentation rate (102 mm/h), hypoalbuminemia (2.4 g/dL), and nephrotic-range proteinuria (12 g/day), with preserved renal function (urea 27 mg/dL and creatinine 0.83 mg/dL). Abdominopelvic CT showed preserved renal parenchyma thickness and good corticomedullary enhancement after contrast administration, with no significant additional findings. Workup for nephrotic syndrome revealed positive anti-PLA2R antibodies (80 RU/mL). Additional investigations were negative, including hepatitis C and HIV serologies, acquired immunity to hepatitis B, negative autoimmune markers (antinuclear antibodies, anti-neutrophil cytoplasmic antibodies, anti-glomerular basement membrane antibodies), as well as negative screening for inherited and acquired thrombophilias.

A diagnosis of PE secondary to primary membranous nephropathy (PMN) was made. Anticoagulation was maintained with enoxaparin and later switched to apixaban after the decision was made not to perform a renal biopsy. Treatment also included diuretics, ACEi, immunosuppression with rituximab (1g), along with prophylaxis with sulfamethoxazole-trimethoprim, which was continued until six months after the last rituximab dose. The patient showed improvement in edema and respiratory symptoms, and was discharged with follow-up plans for ongoing treatment and monitoring.

He received two doses of rituximab three weeks apart and remained on a titrated dose of ACEi. After six months, he had complete resolution of hypoalbuminemia (3.7 g/dL), significant improvement in proteinuria (4.4 g/day), and no decline in renal function. Anti-PLA2R titers were negative. The decision was made to continue anticoagulation until sustained improvement in proteinuria was achieved, alongside ongoing conservative treatment with ACEi.

## Discussion

Although renal biopsy remains the gold standard diagnostic method, we opted not to perform it, considering the presence of anti-PLA2R antibodies, absence of renal impairment, lack of evidence for secondary causes and favorable response to treatment.

The 2021 KDIGO guidelines [6] stratify patients into four risk groups for renal disease progression to guide therapeutic decisions. However, the absence of well-defined thresholds for this stratification, as well as the requirement for ongoing clinical assessment, makes it difficult to determine risk at the time of diagnosis [1]. Nevertheless, in patients with high or very high risk of disease progression, immunosuppressive therapy should be initiated promptly, whereas it may be delayed by three to six months in patients with low or moderate risk [1, 6, 8].

Despite the difficulties, our patient could be considered as high or even very high risk, given the presentation with life-threatening pulmonary embolism, and immunosuppression with rituximab was initiated, since renal function remained stable during hospitalization.

In very high-risk cases, standard therapy typically consists of cyclophosphamide in combination with glucocorticoids [1,6]. To date, alkylating agents remain the only treatment with proven efficacy in preventing end-stage kidney disease and death [8]. Nonetheless, their use has been increasingly delayed or avoided because of the risk of significant adverse effects. Consequently, B-cell-depleting agents and calcineurin inhibitors are now preferred in most cases as first-line treatment [1,2,8].

In this case, prolonged immunosuppression was not required due to the excellent clinical response, with improvement in proteinuria, resolution of hypoalbuminemia, and absence of circulating anti-PLA2R. Although controversial, the decision was made to continue anticoagulation given the severity of the initial presentation, persistence of nephrotic-range proteinuria, and absence of additional bleeding risk factors.

## Conclusions

PMN typically presents with an indolent course but may manifest with severe complications of nephrotic syndrome, as demonstrated in this clinical case. The discovery of specific antibodies has facilitated diagnosis - potentially allowing for the avoidance of renal biopsy - as well as risk stratification for disease progression and monitoring of treatment response. Despite the strong association between PLA2R antibodies and PMN, it remains essential to exclude secondary causes, with renal biopsy still warranted when necessary.

Although some cases may follow a benign course, early initiation of conservative therapy and close clinical monitoring are crucial to prevent disease progression and to assess the need for immunosuppressive treatment.

## References

[1] Wang M, Yang J, Fang X, Lin W, Yang Y (2024). Membranous nephropathy: pathogenesis and treatments. MedComm (2020).

[2] Keri KC, Blumenthal S, Kulkarni V, Beck L, Chongkrairatanakul T (2019). Primary membranous nephropathy: comprehensive review and historical perspective. Postgrad Med J.

[3] Alok A, Yadav A (2025). Membranous nephropathy. In: StatPearls [Internet].

[4] Efe O, So PN, Anandh U, Lerma EV, Wiegley N (2024). An updated review of membranous nephropathy. Indian J Nephrol.

[5] Parker K, Ragy O, Hamilton P, Thachil J, Kanigicherla D (2023). Thromboembolism in nephrotic syndrome: controversies and uncertainties. Res Pract Thromb Haemost.

[6] Kidney Disease: Improving Global Outcomes (KDIGO) Glomerular Diseases Work Group (2021). KDIGO 2021 Clinical Practice Guideline for the Management of Glomerular Diseases. Kidney Int.

[7] Chertow GM, Heerspink HL, Mark PB (2024). Effects of dapagliflozin in patients with membranous nephropathy. Glomerular Dis.

[8] Teisseyre M, Cremoni M, Boyer-Suavet S (2022). Advances in the management of primary membranous nephropathy and rituximab-refractory membranous nephropathy. Front Immunol.

